# Synchronous Seminoma in Abdominopelvic and Inguinal Testes: A Rare Presentation with Unusual Morphology

**DOI:** 10.1155/2017/6179861

**Published:** 2017-02-14

**Authors:** Neha Prabhakar, Bhawna Sethi, Seema Nagger, Arun Saxena

**Affiliations:** Department of Pathology, Faculty of Medicine and Health Sciences, SGT University, Budhera, Gurgaon, Haryana, 122505, India

## Abstract

The development of testes occurs in the abdomen during fetal life, after which they migrate into the scrotal sacs during the third trimester. During their descent, they may get arrested anywhere along the tract. Risk of testicular cancer is higher in patients with undescended testes, abdominal testis being more prone than inguinal. Seminoma is the commonest cancer in undescended testis. However, synchronous seminoma involving bilateral cryptorchid testis is rare. Present case is uncommon due to synchronous involvement of abdominopelvic and inguinal testes in extended age. It also exhibited unusual morphology with marked heterogeneity grossly as well as microscopically, instead of a common homogenous appearance.

## 1. Introduction

The testis may get arrested anywhere along its tract (cryptorchidism) or may migrate into an abnormal position (ectopic testis), during its descent after development in the abdomen. The commonest sites of undescended testis are high scrotal, canalicular, and abdominal ones. Cryptorchidism is encountered in 1% of the boys and is the most significant risk factor for testicular cancer [1]. The incidence of testicular tumor is 11 times more in inguinal testes and 50 times more in intra-abdominal testes [2]. Testicular tumors comprise 1% of all malignancies in males [3]. They may be germ cell tumors or sex-cord tumors. Germ cell tumors are further classified as seminomas and nonseminomas. Seminomas constitute about 40% of all germ cell tumors [4] and are the commonest cancer in undescended testis. However, synchronous seminoma involving bilateral cryptorchid testis is rare [3]. The present case is still the rarer presentation of seminoma in a 56-year-old male, with a synchronous involvement of abdominopelvic and inguinal testes and exhibiting unusual morphology with marked heterogeneity.

## 2. Case Presentation

**Figure 1:**
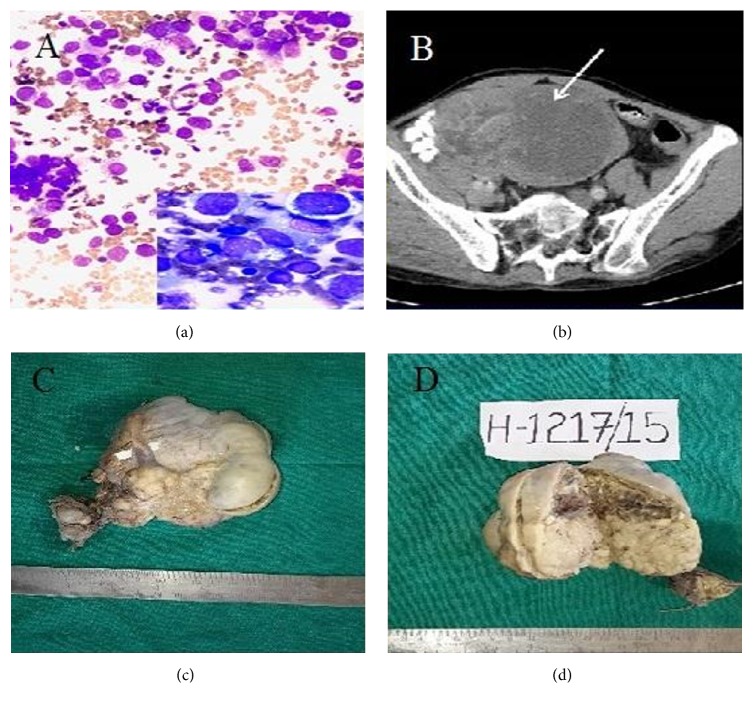
(a) Smear depicting dispersed tumor cells admixed with lymphoplasmacytic infiltrate. (b) CECT depicting heterogeneously enhancing suprapubic and inguinal masses. (c) Gross specimen showing bosselated external surface of the larger mass with attached vas deferens and smooth encapsulated smaller mass. (d) Cut section of masses with more variegated appearance of the larger mass.

A 56-year-male presented with abdominal lump and dragging pain for two years. He was married but had no children. Local examination revealed a lump measuring 10 × 8 × 7 cm in suprapubic region. Ultrasound abdomen revealed a hypoechoic mass with large cystic areas in the suprapubic region. It was abutting the right rectus muscle and was extending into the right inguinal region. Rests of the abdominal organs were normal. Possibility of intra-abdominal tumor/desmoid (since it appeared to be adherent to abdominal wall) was given. Fine needle aspiration from lump showed dispersed tumor cells with round nucleus, large prominent nucleoli, and vacuolated cytoplasm in tigroid background. Many lymphocytes, plasma cells, and occasional epithelioid cells were present [Figure 1(a)]. Occasional mitotic figures were observed. The possibility of germ cell tumor favoring seminoma was kept and detailed workup of the patient was advised. The physical examination showed empty scrotal sacs. Contrast-enhanced computed-tomography revealed two heterogeneously enhancing masses with large cystic areas. The larger mass, in the suprapubic region, was abutting the right rectus muscle. The smaller mass, lying close to the larger mass, was extending to the right inguinal region [Figure 1(b)]. Serum beta-human chorionic gonadotropin, alpha-fetoprotein, testosterone, estradiol, and anti-Mullerian hormone were normal, lactate dehydrogenase was increased, testosterone was mildly reduced, and the karyotype was XY.

**Figure 2:**
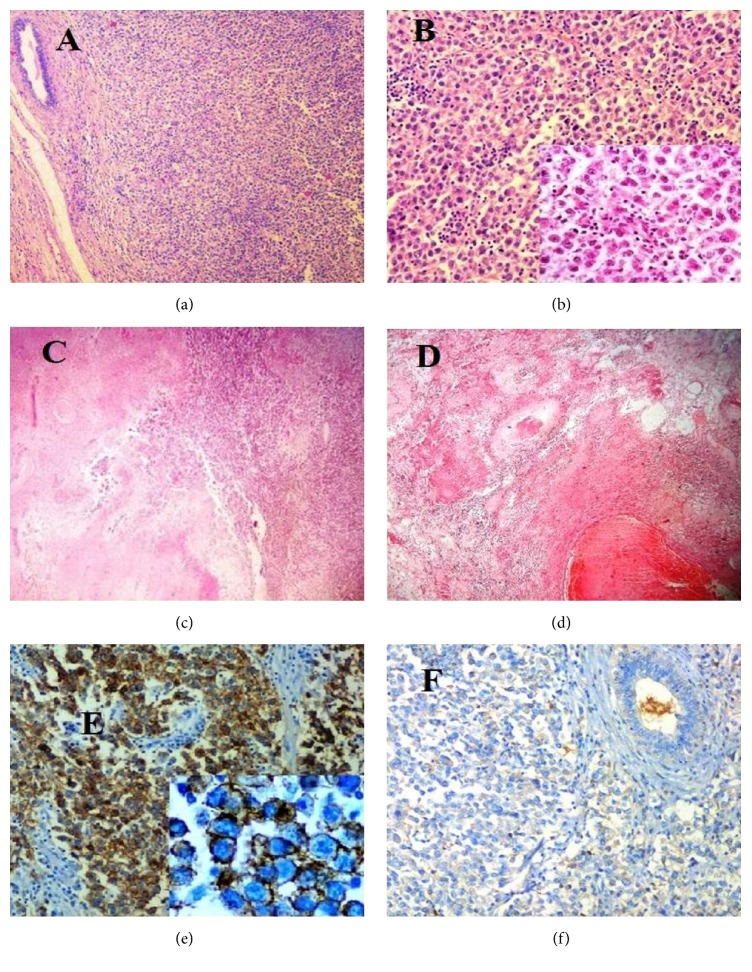
(a) Nests and sheets of tumor cells with a tubule at periphery. H&E, 40x. (b) Tumor cells interspersed with lymphoplasmacytic infiltrate with focal presence of pleomorphic cells. H&E, 100x. (c & d) Areas of hemorrhage, necrosis, and cystic change. (e) Tumor cells positive for CD117 and (f) negative for CD30.

Excision of the lumps was done. The larger mass measured 9 × 8 × 7 cm and the smaller one measured 3 × 2.5 × 2 cm. Both of them were encapsulated. External surface of larger mass was bosselated and had attached vas deferens measuring 4 cm in length. The epididymis, rete testis, and normal testis were not identifiable grossly. Cut surface showed variegated appearance. The smaller one had smooth external surface with relatively less heterogenous cut surface [Figures 1(c) and 1(d)]. Microsections examined from both the masses revealed a cellular neoplasm comprising pleomorphic, vacuolated polygonal tumor cells arranged in nests and sheets separated by fibrous septa. The septa showed infiltration by lymphoplasmacytic cells along with presence of occasional granuloma. Occasional bimultinucleated tumor cells were also seen [Figures 2(a) and 2(b)]. The areas of hemorrhage, cystic change, and necrosis were also observed, the hemorrhage and cystic change being more pronounced in the larger mass [Figures 2(c) and 2(d)]. Lymphovascular tumor emboli were present. A few rudimentary mesonephric duct elements were observed at the periphery of masses. Only a portion of ductus deferens was infiltrated by the tumor; the cut end was free. The tunica albuginea was involved. The cytoplasm of tumor cells showed positivity with periodic acid Schiff stain. Immunohistochemistry was performed on two blocks from cystic areas. It revealed diffuse positivity for CD117 and negativity for CD30 [Figures 2(e) and 2(f)], serum alpha-fetoprotein (AFP), and beta-human chorionic gonadotropin (*β*-hCG). Only focal positivity (approximately 3%) for cytokeratin (CK) was noted.

## 3. Discussion

Testicular tumors comprise about 1% of all malignancies in males. It is however the most common tumor in males between 15 and 35 years [5]. Germ cell tumors account for approximately 94–96% of the testicular tumors. They are divided into seminomas and nonseminomas. Seminomas are the commonest pure germ cell tumors [6]. Nonseminomatous tumors include yolk sac, embryonal, teratoma, and choriocarcinoma. The distinction between seminoma and nonseminoma is the main factor which directs the treatment [5]. The important risk factors for testicular tumors are cryptorchidism, a previous testicular tumor, family history suggestive of testicular tumors, and somatosexual ambiguity syndromes [3]. Subfertility and infertility have increased risk of developing testicular cancer. Germ cell neoplasia in situ (GCNIS) is an important precursor [7]. An association of testicular cancer with low birth weight and small for gestational age babies and hypospadias has also been supported. Other, less consistent factors include low birth order, high maternal age, neonatal jaundice, and retained placenta. All testicular germ cell tumors, including their precursor GCNIS, are aneuploid. Seminoma and GCNIS cells are hypertriploid, while the tumour cells of nonseminoma, irrespective of their histological type, are hypotriploid [7, 8]. The most common structural cytogenetic abnormality in seminoma is presence of isochromosome 12p. Some patients lack the isochromosome 12p but have other structural chromosomal abnormalities. The pathogenesis of germ cell tumors has been linked to primordial germ cells. The receptor tyrosine kinase (c-kit) is necessary for migration and survival of primordial germ cells. It is expressed in germ cell neoplasia in situ and seminomas. The mutation frequency of c-kit exon 17 has been found to be significantly higher in bilateral synchronous seminomas [3, 7].

Patients with seminoma of testis typically present with a painless testicular enlargement/mass, sometimes associated with an ill-defined aching sensation in the lower abdomen, inguinal region, or scrotum [5, 8]. Some patients with seminoma are asymptomatic. Gynecomastia, exophthalmos, and infertility are rare presenting symptoms. Elevated serum PLAP and hCG are seen in 40% and 10% of the patients, respectively. Pure seminoma may show mild elevation of serum hCG levels because of the syncytiotrophoblastic giant cells. However, significant elevation in serum AFP level virtually excludes a diagnosis of pure seminoma, even though microscopic evaluation may show only the seminomatous component. NSE is another marker, increased in patients with metastatic seminoma, which normalizes after chemotherapy [8].

Fine needle aspiration smears show dispersed population and loose clusters of cells with fragile cytoplasm and round to ovoid nuclei with nuclear smudging. The nuclear chromatin is irregular with presence of single/multiple moderate sized nucleoli (smaller than embryonal carcinoma). Mitotic figures may be found. Variable numbers of lymphocytes and plasma cells may be intermingled with the tumor cells. A few cases have shown epithelioid histiocytes as well [9, 10]. On gross examination, the tumor is usually well-circumscribed, homogeneous, firm mass, frequently gray-white, lobulated, and bulging. Hemorrhage and necrosis are uncommon but may be seen in large tumors. The average size is 5 cm, with rare cases exceeding 10 cm [6, 8, 11]. Microscopic examination shows large, uniform tumor cells arranged in sheets, nests, or cords. Tubular, reticular, cystic, and cribriform patterns have also been reported. Areas of typical seminoma are always present in tumors with such variations. The tumor is separated into lobules by a supporting stroma, which contains a variable number of lymphocytes. The extent of lymphocytic (T cells) infiltration varies from tumor to tumor and within different parts of the same tumor. Marked lymphoplasmacytic infiltrate or granulomatous inflammation may also be present, which may even overwhelm the tumor [12]. The stroma also varies in amount and appearance. Rarely, seminiferous tubules can be entrapped within the tumor, usually at the periphery with a granulomatous reaction. Staining by PAS with and without diastase demonstrates glycogen in the cytoplasm of the tumor cells. Immunostaining for placental alkaline phosphatase (PLAP), C-kit (CD117), Angiotensin 1-converting enzyme, and D2-40 show diffuse positivity. Vimentin can be positive, but EMA and CD 30 are negative in most seminomas. Cytokeratin is usually negative but can be focally positive in tumor cells. The syncytiotrophoblastic giant cells are positive with cytokeratin and hCG [6, 8, 13]. Studies have shown that germ cell tumors express several genes that are required for the maintenance of “stemness” of embryonic stem cells. High expression levels of a cadre of such genes like SOX2, growth differentiation factor-3 (GDF3), TDGF, EBAF, and FGF4 have been observed in embryonal carcinoma, but not in seminoma. In contrast, cooverexpression of STELLA, NANOG, and OCT4 has been found in both seminoma and embryonal carcinoma [14].

The concerned case presented with abdominal mass and dragging pain. The serum markers were normal. Classical cytological features helped reach the diagnosis, though a few unusual features like focal presence of pleomorphic cells and few multinucleated tumor cells were also observed. Grossly, the tumor showed variegated gross appearance with large cystic areas, as noted in large tumors by other authors. Microscopically, there were marked pleomorphism, atypia, and mitosis. The tumor was interspersed with lymphoplasmacytic infiltrate as well as epithelioid granulomas. It revealed diffuse positivity for CD117, only focal positivity (approximately 3%) for cytokeratin (CK), and negativity for CD30 (Figures 2(e) and 2(f)), serum alpha-fetoprotein (AFP), and beta-human chorionic gonadotropin (*β*-hCG).

The pure seminoma needs to be differentiated from malignant lymphoma and mixed germ cell tumor with embryonal carcinoma and/or endodermal sinus tumor and/or choriocarcinomatous components and spermatocytic seminoma. The lymphoma shows interstitial infiltration of tumor cells between the seminiferous tubules and lacks fibrosis or granulomatous reaction. The cells are positive for leukocyte common antigen (LCA) in contrast to seminoma. Embryonal carcinoma is usually seen in younger age. Microscopically, it shows greater cellular pleomorphism and more brisk mitotic activity. The tumor cells are positive for cytokeratin and CD 30 and negative for D240, as against seminoma [8, 13]. Pure endodermal sinus tumor is usually seen in children and is more likely to be arranged in reticular, myxoid, and microcystic patterns. It shows presence of basement membrane-like material between tumor cells (parietal yolk sac differentiation). Cytokeratin and AFP immunostaining are helpful in differentiating this tumor from seminoma. Although spermatocytic seminoma is relatively common in patients over 50 years of age it consists of round cells with marked polymorphism and presence of three-cell population and is not associated with fibrovascular septa, lymphocytic infiltration, or granulomatous reaction. Due to presence of occasional giant cells, necrosis and hemorrhage, choriocarcinoma was excluded due to lack of typical biphasic appearance, abortive villous architecture, and hCG immunostaining. Due to high mitotic rate, the possibility of anaplastic seminoma, which is characterized by increased mitotic activity (≥3 mitoses per high-power field) and nuclear pleomorphism was considered. However, since it has not attained the separate nomenclature, the diagnosis of seminoma was made [8].

The most important prognostic factor for seminoma is the clinical stage at presentation. Rest of the pathologic parameters, including the intensity of lymphoplasmacytic infiltration, the degree of granulomatous reaction, tumor necrosis, fibrosis, invasion, and interstitial cell hyperplasia, do not correlate with survival. However, a tumor size greater than 6.0 cm has been correlated with a higher rate of relapse [8]. Management of the present case was comparatively tougher due to lack of literature and rarity of the presentation. Bilateral germ cell tumors of testis are rare with incidence ranging from 1% to 5%. They are either synchronous or metachronous. Synchronous bilateral primary germ cell tumor of the testis is rare and its association with bilateral cryptorchidism is even rarer. Only few cases have been reported [Table 1] [4, 15–23].

In the concerned case, bilateral orchiectomy was done for diagnostic as well as therapeutic purposes. The tumor was staged as IB [24]. But since it has been proposed that bilateral seminomas have higher tumor burden and they should be treated with prophylactic para-aortic lymph node irradiation or one and two cycles of adjuvant chemotherapy [3], the case has been referred for further management.

## 4. Conclusion

Possibility of seminoma should be considered in adult male presenting even with heterogenous (radiological and morphologically) abdominal mass for prompt management.

- A thorough examination of genitals is advised in such cases.
- Nonseminomatous elements should be ruled out, especially if the tumor is heterogenous.

## Figures and Tables

**Table 1:** 

Author	Age & sex	Location	Size	TM (LDH *β*-hCG *α*-AFP)	Gross findings	Histomorphology	Stage	Treatment	F/U^*∗*^
Darabi and Barzegarnejad (2004)	23 M	Intra-abdominal (pelvic)	Big (NM)	NM, Nor, ↑	NM	Classic seminoma and embryonal carcinoma	II	Bilateral orchidectomy + RT	NM
Agrawal et al. (2010)	23 M	Intra-abdominal (both)	Big (NM)	Nor	Hetero (hemorrhage + necrosis)	Pure seminoma	IA	Surgery & 4CT (BEP)	5 yr
García Morúa et al. (2010)	44 M	Inguinal and pelvic cavity	15 & 10	601, 11.8, 5.08	Hetero (solid & cystic)	Pure classic testicular seminoma	I & II	Bilateral orchiectomy (NM further)	NM
Kumar et al. (2012)	30 M	Intra-abdominal (left iliac fossa and post to UB)	6.5 & 7.4	1257 0.38 2.02	No areas of hemorrhage/necrosis	Poorly diff. sem	I	Surgery & 4CT (BEP)	8 moths
Singh et al. (2012)	26 M	Left iliac fossa and lumbar region (ext into inguinal canal)	10 & 4.0	378, 1.49, 3.26	Hetero (cystic areas and calcification)	Seminoma and GCNIS	IA	Surgery & 3CT (BEP)	NM
Seetharam et al. (2014)	28 M (heterosexual)	Intra-abdominal (lumbar region)	13.4 & 9.6	2890, Nor, Nor	Homogenous	Pure seminoma	I	Surgery & 4 CT (BEP)	6 mths
Ghartimagar et al. (2014)	40 M	Abdominopelvic	12.2 & 10.7	519, 1.1, 9.2	Homogenous	Classical seminoma	NM	Surgery & 4 CT (BEP)	8 mths
Rao et al. (2015)	37 M	Bilateral inguinal	7.0 & 1.0	210, 4.07, 0.2	Solid, grey white to yellow, homogenous	Seminoma	I	Bilateral orchiectomy	NM
Afsar et al. (2016)	36 M	Bilateral inguinal	5.5 & 1.0	NM	Yellow homogenous	Seminoma	NM	Bilateral orchiectomy	NM
Present case (2016)	56 M	Intra-abdominal and inguinal	9.0 & 3.0	790, 2.6, 2.8	Hetero	Seminoma with anaplastic features	IB	Bilateral orchiectomy & CT	9 mths

TM: tumor markers.

LDH: lactate dehydrogenase (U/L).

*β*-hCG: beta-human chorionic gonadotrophin (mIU/ml).

*α*-AFP: alpha-fetoprotein level (ng/ml).

F/U^*∗*^: follow-up (with no recurrence).

NM: not mentioned.

Nor: normal.

Hetero: heterogenous.

CT: chemotherapy.

BEP: bleomycin, etoposide, and cisplatin.
